# RAFnet: SAR Image Autofocusing via Range-Aware Attention and Multi-Scale Loss

**DOI:** 10.3390/s26134270

**Published:** 2026-07-04

**Authors:** Hua Wu, Yan Liu, Yunbai Qin, Haoran You, Zhuoxiang Lin

**Affiliations:** 1 School of Electronic and Information Engineering, Guangxi Normal University, Guilin 541004, China; hwu@stu.gxnu.edu.cn (H.W.); youhaoran@stu.gxnu.edu.cn (H.Y.); linzx@stu.gxnu.edu.cn (Z.L.); 2 College of Electronic and Information Engineering, Shenzhen University, Shenzhen 518000, China; 2552042003@mails.szu.edu.cn

**Keywords:** synthetic aperture radar (SAR), autofocus, attention mechanism, deep learning

## Abstract

Platform motion errors degrade SAR image quality in terms of severe defocusing and azimuth blurring. We propose a Range-aware Autofocus Network (RAFnet) by embedding a novel range-aware attention module into a progressive autofocus framework. The module exploits 1-D azimuth pooling to compress spatial features and extract high-SNR scattering components from the range dimension. Such features are further enriched via a light cross-channel interaction. To facilitate coarse-to-fine hierarchical learning, we develop a progressive multi-scale entropy loss which jointly optimizes the entire network. Experimental results on real SAR data show that the proposed approach captures high-level phase fluctuations accurately and effectively suppresses raw phase deviations and sidelobes. Quantitative results show that combining the attention module with multi-scale loss achieved a global spatial entropy of 9.8567 and contrast of 5.0091 in focused images. By extracting more accurate focus-oriented feature representations, we provide an effective solution for high-quality SAR auto-focusing.

## 1. Introduction

Synthetic aperture radar (SAR) has been widely used in both military surveillance and civil tasks such as disaster monitoring and response due to its all-weather, day-and-night imaging capability and 2-D localization [1,2]. However, due to the non-linear phase error introduced by the motion instabilities on the radar platform, the image can rapidly become distorted due to complex azimuth blurring or geometric distortions. These degradations not only mask the target contour and erase fine details of the texture, but also significantly decrease the spatial resolution and localization performance [3,4]. Consequently, autofocusing techniques have been extensively studied as a key technique to achieve image quality. The conventional autofocus methods focus more on non-parametric phase estimation and parametric modeling. The classic phase gradient autofocus (PGA) algorithm [5], and its popular variants, linear unbiased minimum variance PGA (PGA-LUMV) [6], and maximum likelihood PGA (PGA-ML) [7], compensate for the arbitrary random non-linear errors but rely heavily on precise extraction and centering of major dominant scatterers in the scene [8]. While the general map drift algorithm (MDA) relies less on isolated dominant scatterers, its correction ability is limited by low-order polynomial fitting, which will make it ineffective with high-order motion errors [9]. Unlike the existing methods based on local pixel phases or geometric relationships, normalized minimum entropy algorithms (NMEA) [10] focus on global image quality scores. Examples include image sharpness [11,12], contrast [13,14], information entropy [15,16,17], as well as peak signal-to-noise ratio (PSNR) and structural similarity index measure (SSIM) [18]. This approach transforms phase correction into an optimization problem of a target cost function, and thus progressively improves image quality by updating its phase error incrementally. Nevertheless, the extensive iterative searching process results in a severe computational burden, particularly in terms of prohibitive execution time when processing large-scale high-resolution data. Traditional algorithms like PGA-ML take an average of 163.71 ms to process each image, while the method proposed in this paper only takes 1.73 ms, which greatly saves computation time while still maintaining focusing accuracy.

With the development of deep learning, using neural networks to handle SAR autofocus problems has gradually become a popular research direction. Liu et al. [19] first proposed an unsupervised autofocus architecture, AFnet, based on convolutional neural networks, which relies purely on data to eliminate dependence on ground truth images. Subsequently, PAFnet [19] adopted a layer-by-layer focusing strategy to improve computational efficiency, but when performing spatial feature aggregation, it treats all range cells equally, making it unable to distinguish between scatterers with relatively strong signal-to-noise ratios and background clutter. This causes the network’s azimuth phase estimation in complex scenes to be easily contaminated by speckle noise, resulting in low focusing accuracy under severe nonlinear errors. Ref. [20] proposed a Sparse Autoencoder Network (SAENet), which maps SAR echoes to the image domain through an encoder and reconstructs the echo signals via a decoder to achieve end-to-end feature learning. However, this approach is mainly applied in sparsity-driven SAR imaging scenarios. In addition, its global matrix mapping structure has serious memory overflow issues when handling large and high-resolution images. SPA-GAN [21] attempts to integrate a generative adversarial network with traditional parameterized autofocus models. Although this method uses deep learning to obtain better parameter estimation, it is essentially based on preset physical models and struggles to cope with extremely complex model mismatch and distortion problems.

In recent years, with the development of attention mechanisms, it has been widely used not only in the field of traditional computer vision, but also in radar and microwave tasks such as magnetic resonance imaging (MRI) reconstruction [22], astronomical image restoration [23], SAR image object detection [24,25,26,27], and SAR image despeckling [28]. The method in [29] mainly strengthens the anisotropy of azimuthal features through a lightweight attention module. Reference [30] introduces the Restormer module based on the Transformer architecture, enabling global feature interaction through a cross-channel self-attention mechanism. However, by peeling off the shell of its extraction of visual semantics, it can be seen that mathematically speaking, the attention mechanism is a means based on data-driven adaptive screening and weighting features [31], which amplifies valid features and strongly suppresses invalid noise through the analysis of the correlation of the internal structure of the input tensor. In the deep learning-based SAR self-focusing task, the network input is often a defocused image that is heavily polluted by strong coherent speckle noise and complex background clutter. From the perspective of parameter estimation, this is a typical source of heterogeneous quality; that is, not all spatial pixels will make a positive contribution to solving azimuth phase errors. If a large area of pure clutter region and the effective target are equally weighted into the network for global aggregation, it is equivalent to injecting huge interference noise into the phase estimator and deteriorating the signal-to-noise ratio of the effective signal. Therefore, this paper introduces attention into the SAR self-focusing model, which is not actually aimed at extracting high-level semantics, but only physically maps the attention mechanism as a data quality filter, which can block the influence of clutter noise on phase solution from the root to the effective structural adaptive weighting containing high signal-to-noise ratio. The method proposed in this paper achieved a PSNR of 25.3870 dB, which was the best performance among the comparisons above. This metric also strongly confirms the advantages of our attention mechanism in suppressing background clutter and increasing the signal-to-noise ratio of the effective signal.

On the whole, most of the existing feature extraction methods in the network treat all range elements equally and lack the characteristics of finely perceived SAR image range scattering. As a result, it is difficult to identify and extract the target area with strong signal-to-noise ratio in severe background clutter, and the model therefore struggles to obtain better phase and focusing results under conditions of extreme nonlinear error. To solve the above problems, this paper proposes a feature enhancement scheme based on single-dimensional range-aware attention based on the existing unsupervised deep learning network, and designs a new loss function to coordinate with the existing network to break the bottleneck of clutter interference in the existing network and achieve high SAR image self-focusing results. The main contributions of this paper are as follows.

1. A new range-aware attention module is proposed. During training, this method only selects features obtained from range, serving as a pre-processing data quality filter, adaptively weighting high signal-to-noise ratio targets while suppressing background clutter to filter the range gate. This results in a significant enhancement of the baseline PSNR from 13.5987 dB of the original defocused image to 25.3870 dB, such that more reliable features can be used for describing the phase when estimating the azimuth direction;
2. A multi-scale self-focusing loss function is constructed to be used in conjunction with feature enhancement: when image entropy is highly non-convex and prone to local optima, the coarse-to-fine gradient distribution helps the network focus on topology, effectively ensuring the model’s convergence accuracy and stability under severe phase disturbances;
3. The algorithm in this paper achieves good focusing performance on real SAR datasets. Comparisons show that, compared with representative algorithms such as PGA, NMEA, SAENet, and PAFnet, the proposed algorithm achieves satisfactory results in various indicators, including image entropy and contrast.

## 2. Materials and Methods

### 2.1. Fundamental Background

Platform motion errors, propagation effects, and system instability are the main causes of phase degradation in SAR images. For medium-resolution strip-map SAR, the residual RCM after range cell migration correction (RCMC) over a certain range is often neglected. Range block processing can ensure consistency of phase errors within a block [32]. Therefore, the complex 2-D autofocus is simplified to a one-dimensional (1-D) phase compensation problem. Let $I{\;\in \;C}^{N_{a}\times N_{r}}$ be the degraded image to be processed, where $N_{a}$ and $N_{r}$ represent the number of sampling points in the azimuth and range directions, respectively. This autofocus problem can be described as:

(1) $$Y_{n_{a}n_{r}}=\frac{1}{N_{a}}\sum_{k=0}^{N_{a}-1}X_{kn_{r}}\exp\{-j\phi_{k}\}\exp\left\{j\frac{2\pi }{N_{a}}kn_{a}\right\}$$

Here, $Y_{n_{a}n_{r}}$ represents the time-domain focused image after compensation. X is the range-Doppler domain data of the degraded image I, which can be obtained by performing a Fourier transform of I in the azimuth direction. The key point of autofocus is to estimate the corresponding azimuth phase error $\phi_{k}$ from the defocused data X. To achieve a parametric description of the phase error, a polynomial model is used to model the azimuth phase error $\phi_{k}$. According to the theory of Taylor series expansion, the residual phase error $\phi_{k}$ can be considered as a linear combination of higher-order polynomial terms.

(2) $$\phi \left(k,\alpha \right)=\sum_{i=2}^{Q}\alpha_{i}f_{k}^{i}$$

Here, $f_{k}$ represents the normalized frequency of the *k*-th Doppler frequency bin, and $\alpha ={\left[\alpha_{2},\alpha_{3},\ldots ,\alpha_{Q}\right]}^{T}$ is the vector of polynomial coefficients to be estimated. Under this parameterized framework, achieving better autofocus regression results depends on the accuracy of the coefficients α, which determine whether the final phase term $\phi_{k}$ can be recovered. Since traditional convolution is prone to interference from background clutter in large-scale and complex scenarios, this study employs range-aware attention. This article uses range-aware attention to give more weight to effective scattering structures with a high signal-to-noise ratio (specifically, targeting a PSNR exceeding 25 dB in our network design). During backpropagation, this module acts as a dynamic weight mask to explicitly suppress gradients from background clutter, stably guiding the phase estimation to converge toward correct values.

**Figure 1:**
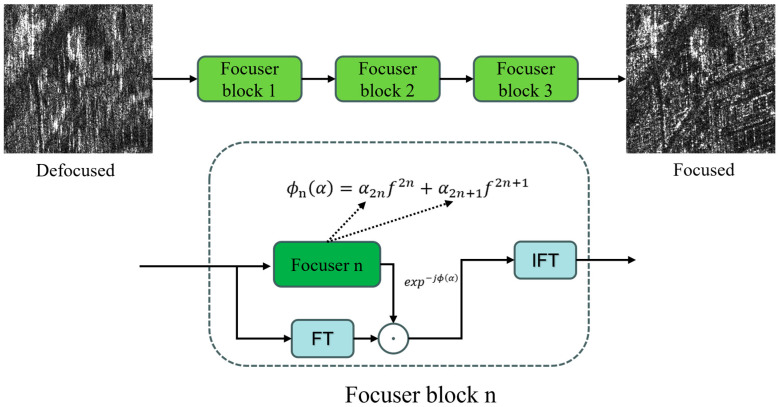
Flowchart of the focusing process.

The overall flowchart of the proposed focusing process is illustrated in Figure 1. The defocused initial image is input into a cascaded correction framework containing three levels of focusers. Each focuser regresses a set of phase coefficients corresponding to higher-order polynomials through an internal feature extraction network and compensates the current feature map according to these coefficients. The compensated output at the current level serves as the input to the next-level focuser, thereby progressively removing residual errors. A single focuser can be set to estimate and correct phase errors up to two orders. Since low-order motion errors do not cause defocus, estimation starts from the quadratic term. After three cascaded levels, the residual errors of orders {2,3}, {4,5}, and {6,7} can be corrected sequentially, where the first focusing module is used to correct 2nd- and 3rd-order errors, the second focusing module handles 4th- and 5th-order errors, and the third focusing module corrects 6th- and 7th-order errors. Each focusing module has the same network structure, but their weights are not shared so they can meet the correction needs for errors of different orders, ultimately achieving finer calibration of high-order phase distortions up to the 7th order and producing an excellent fully focused output image.

### 2.2. Overall Network Architecture

**Figure 2:**
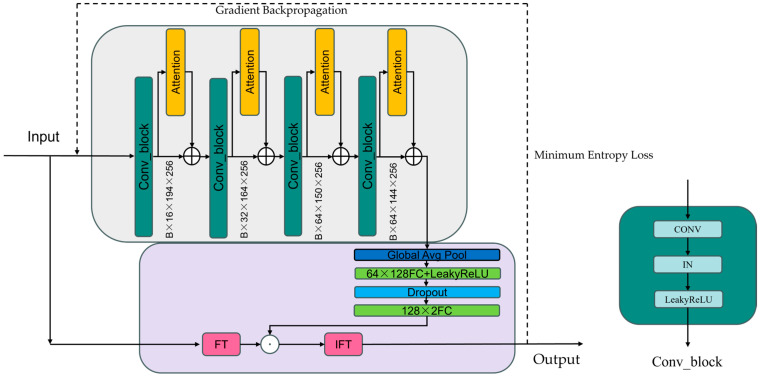
Detailed architecture of the individual focuser.

The detailed structure of a single focus unit is shown in Figure 2. The focus unit consists of four cascaded feature extraction blocks and a parameter vector regression head. Each feature extraction block contains a convolutional block and a parallel attention module. The convolutional block includes a 2D convolutional layer, a normalization layer (IN) [33], and a LeakyReLU [34] activation function. The attention module serves as an enhancement branch, using spatial correlation along the range dimension to help suppress background clutter. The output is element-wise, fused with the main convolutional features via a residual connection [35], which better enhances the network’s perception of areas with strong scattering points. After four stages of feature extraction, the original feature maps are compressed into a one-dimensional feature vector through a global average pooling layer and fed into a regression head composed of two fully connected layers for integration. The first fully connected layer handles high-dimensional features, with an intermediate Dropout layer [36] with a rate of 0.5 integrated to enhance generalization ability and prevent overfitting. The final fully connected layer maps to the output of the final phase coefficients.

### 2.3. Range-Aware Attention 

Traditional convolution operations mostly perform feature equalization extraction for local receptive fields, making it difficult to deal with strong clutter interference in complex autofocus scenarios. In SAR imaging, all range cells within a data block have the same azimuth phase error, but in practical complex scenes, not all of them are high signal-to-noise ratio effective structures. Therefore, the purpose of introducing range-aware perception is not to estimate range errors, but to use spatial correlation for weighting. This module dynamically suppresses pure clutter without definite phase history by transforming the weights assigned to features across the range dimension and amplifies the weighting of effective range cells with strong scattering targets, greatly enhancing the network’s sensitivity to high signal-to-noise ratio effective features.

**Figure 3:**
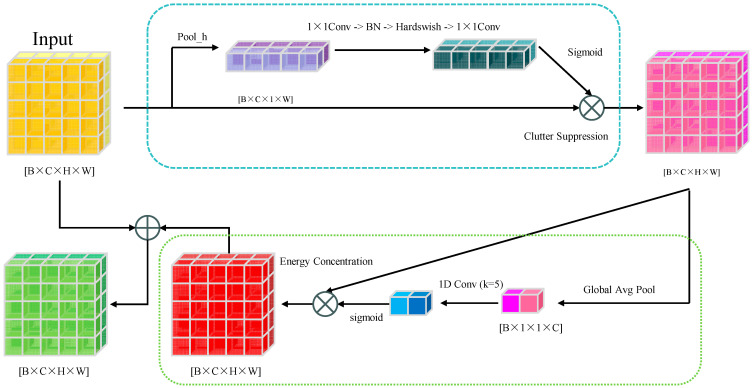
Range-aware attention module.

As schematically illustrated in Figure 3, the designed attention module is structured by cascading a range spatial branch with a channel attention branch, whose terminal output is fused with the pristine input features via a global residual connection. Consider a given input feature tensor $X\in R^{B\times C\times H\times W}$, where B, C, H, and W denote the batch size, channel number, azimuth dimension, and range dimension, respectively. To exploit the spatial correlations along the range direction, the module performs 1-D pooling on the input along the azimuth axis, compressing the global spatial information into orientation-aware feature maps with a dimension of B × C × 1 × W. Subsequently, these feature maps are routed into a feature transformation layer comprising a 1 × 1 convolution, a batch normalization (BN) layer [37], a Hardswish activation function [38], and a secondary 1 × 1 convolution. Finally, a Sigmoid activation function [39] is applied to yield non-linear spatial weights. This weight matrix is element-wise multiplied by the pristine input X, producing the intermediate feature tensor enhanced along the spatial dimension.

(3) $$\tilde{X}=X⊗\sigma \left(F_{w}\left({\mathrm{Pool}}_{h}\left(X\right)\right)\right)$$

${\mathrm{Pool}}_{h}\left(\cdot \right)$ represents a one-dimensional average pooling operation along the azimuth direction, and $F_{w}\left(\cdot \right)$ represents the feature transformation operator composed of a 1 × 1 convolution, batch normalization, and the Hardswish activation function. $\sigma \left(\cdot \right)$ is the Sigmoid function; ⊗ denotes element-wise multiplication; and $\tilde{X}$ is the intermediate feature tensor after spatial weighting. After completing the weighting over the spatial dimension, the intermediate feature tensor enters the channel attention branch to again identify the feature channels that contribute more to phase compensation. In this attention branch, global average pooling is first used to compress the two-dimensional spatial dimensions, resulting in a feature map of size B × C × 1 × 1. To accommodate the subsequent one-dimensional convolution computation, the network reshapes this tensor to obtain a feature map of size B × 1 × 1 × C. Then, a one-dimensional convolution with a kernel size of 5 is used to perform local cross-channel interaction. After that, the Sigmoid function is used to obtain the final channel weight matrix. The channel weight matrix is again applied via element-wise multiplication to the intermediate features, thereby realizing the weighting of channel features.

(4) $$\hat{X}=\tilde{X}⊗\sigma \left({\mathrm{Conv}1D}_{k}\left(\mathrm{GAP}\left(\tilde{X}\right)\right)\right)$$

GAP(⋅) represents the global average pooling operation, and ${\mathrm{Conv}1D}_{k}\left(\cdot \right)$ is a one-dimensional convolution operator with a kernel size k = 5, which implicitly involves the process of dimension rearrangement and restoration. $\hat{X}$ represents the enhanced feature tensor obtained after channel weighting. Finally, the features calibrated by dual attention are connected with the original input tensor X via a residual connection, and the module’s final enhanced feature map is merged element-wise:

(5) $$Y=\hat{X}+X$$

Y is the final output tensor of the attention module with a global residual connection.

### 2.4. Multi-Scale Loss Function

Fundamentally, the autofocusing of SAR images can be construed as an optimization process aimed at seeking the optimal image sparsity and localized contrast [40]. The 2-D spatial entropy of an image serves as an intuitive and high-fidelity metric to mirror this underlying physical attribute: more pronounced defocusing anomalies inherently scatter the radar echoes across the imaging plane, thereby elevating the entropy value; conversely, sharper focusing performance concentrates the energy profiles onto strong scatterers, yielding a minimized entropy. Given the highly non-linear nature of defocusing distortions, brutal direct optimization risks trapping the network into suboptimal local extrema. To comply with the physical tenets of tier-by-tier optimization, this study leverages the minimum entropy algorithm in tandem with the topological characteristics of the cascaded networks, formulating a deep supervision loss function driven by minimum entropy. To circumvent the inherent limitation of conventional architectures—where constraints are strictly enforced only at the terminal output layer—and to guide the cascaded framework in achieving coarse-to-fine progressive phase compensation, independent loss constraints are seamlessly injected across all three cascaded focusers. By introducing extra intermediate deep supervision signals into the shallow and mid-level layers, this mechanism not only effectively immunizes the feature learning of middle focusers against degradation, but also significantly mitigates the overarching optimization difficulty of the network. The formal mathematical formulation of the total multi-scale joint loss function is prescribed as follows.

(6) $$L_{total}=\lambda_{1}L^{\left(1\right)}+\lambda_{2}L^{\left(2\right)}+\lambda_{3}L^{\left(3\right)}$$

Here, $L^{\left(1\right)}$, $L^{\left(2\right)}$, and $L^{\left(3\right)}$ signify the minimum entropy autofocusing losses calculated from the output results of the first, second, and terminal focusers, respectively. The hyper-parameters $\lambda_{1}$, $\lambda_{2}$, and $\lambda_{3}$ constitute the balancing weight coefficients embedded within the multi-scale deep supervision framework. To balance the coarse-level phase compensation guidance during the early operational stages of the network with the fine-grained focusing fidelity of the terminal output, this study strategically scales down the penalty proportions of the intermediate estimation processes. Specifically, the weight coefficients for the preliminary focusers are configured as $\lambda_{1}$ = $\lambda_{2}$ = 0.2, whereas the terminal output stage is assigned the maximum optimization priority with $\lambda_{3}$ = 1.0. For an arbitrary *K*-th focusing stage within the network (K∈1,2,3), assuming its output batch of focused complex-valued images is denoted by $Y^{\left(k\right)}\in C^{B\times 1\times H\times W}$, the formal mathematical formulation of the single-stage minimum entropy loss $L^{\left(k\right)}$ derived from the pixel energy distribution is prescribed as follows:

(7) $$L^{\left(k\right)}=-\frac{1}{B}\sum_{b=1}^{B}\sum_{h=1}^{H}\sum_{w=1}^{W}\frac{{\left|Y_{b,h,w}^{\left(k\right)}\right|}^{2}}{Z_{b}^{\left(k\right)}}\ln\left(\frac{{\left|Y_{b,h,w}^{\left(k\right)}\right|}^{2}}{Z_{b}^{\left(k\right)}}\right)$$

(8) $$Z_{b}^{\left(k\right)}=\sum_{h=1}^{H}\sum_{w=1}^{W}{\left|Y_{b,h,w}^{\left(k\right)}\right|}^{2}$$

Here, B signifies the current training batch size, while H and W correspond to the azimuth and range dimensions of the image, respectively. The notation $Y_{b,h,w}^{\left(k\right)}$ represents the complex-valued pixel intensity located at the spatial coordinate (h,w) for the b-th sample within the k-th stage output. The operator ${\left|\cdot \right|}^{2}$ designates the calculation of the squared pixel intensity, whereas $Z_{b}^{\left(k\right)}$ acts as the total energy normalization factor for the corresponding single SAR image in the spatial domain. During the backpropagation phase, minimizing the overarching multi-scale loss $L_{total}$ continuously drives the output of each sequential module toward a sharper focus characterized by elevated localized contrast and reduced entropy, ultimately achieving high-precision phase error compensation.

## 3. Results

### 3.1. Datasets

The data used in this study [41] were obtained from the Advanced Land Observing Satellite (ALOS) developed by the Japan Aerospace Exploration Agency (JAXA) in fine mode, using a total of 9 scenes of synthetic aperture radar (SAR) raw echo data. Specifically, the data have an azimuth resolution of about 5 m and a ground range resolution of approximately 7 m. These data cover regions such as Vancouver, Xi’an, Hefei, Florida, Toledo, and Silicon Valley. To obtain a rich defocused dataset, we generated azimuth phase errors by simulating equivalent radar velocity estimation errors. To ensure the objectivity and independence of the evaluation results, the raw data were divided into non-overlapping training, validation, and test sets. The training set consisted of 125 defocused images corresponding to 5 base scenes; the validation and test sets each consisted of 50 defocused images corresponding to 2 additional base scenes. During the data preprocessing stage, 256 ×256 complex-valued image patches were extracted by cropping the aforementioned defocused images to meet the network input requirements. Finally, we randomly selected 20,000 image patches from the training data for training, and 8000 image patches each were extracted from the validation and test data for validation and testing.

### 3.2. Implementation Details

All related experiments in this study were conducted on a computer running the Ubuntu 22.04 operating system. The hardware includes an Intel^®^ Xeon^®^ Platinum 8470Q (25 vCPU) and a single NVIDIA RTX 5090 graphics processor with 32GB of memory (NVIDIA, Santa Clara, CA, USA). The software includes the Python 3.12 programming language and the PyTorch 2.7.0 deep learning framework. Additionally, CUDA 12.8 is used to accelerate underlying hardware computations. The batch size and number of training epochs were set to 8 and 500, respectively, and the AdamW optimizer [42] with a weight decay factor of 0.01 was used, with the learning rate updated according to the strategy in [19]. During the training phase, model convergence was monitored by analyzing the training loss curve. To ensure optimal generalization performance and mitigate potential overfitting, the model weights that achieved the lowest loss on the validation set were explicitly selected for the final testing phase and subsequent experimental evaluation. The network and experiments proposed in this paper have been open-sourced at https://github.com/wljwdsa/sar (accessed on 20 June 2026).

### 3.3. Evaluation Metrics

In order to objectively and quantitatively verify the effectiveness of the multi-scale loss function and range-aware attention module proposed in this paper, four metrics, image entropy, image contrast, peak signal-to-noise ratio (PSNR), and structural similarity index measure (SSIM), are used to reflect the focusing quality of SAR images from different perspectives. Image entropy represents the spatial distribution of energy in the image. When the network compensates for phase errors and defocusing distortions are eliminated, the energy of strong scattering points in the image (such as buildings, vehicles, etc.) becomes more concentrated, resulting in a decrease in overall image entropy. When the network finally outputs a complex-valued focused image Y of size H ×W, the calculation formula for the two-dimensional spatial entropy E is as follows.

(9) $$E=-\sum_{h=1}^{H}\sum_{w=1}^{W}\frac{{\left|Y_{h,w}\right|}^{2}}{Z}\ln\left(\frac{{\left|Y_{h,w}\right|}^{2}}{Z}\right)$$

In the equation, h and w represent the pixel indices in the azimuth and range directions, respectively, and $\left|Y_{h,w}\right|$ is the amplitude at that pixel. Z is the image total energy normalization factor, which is calculated as $Z=\sum_{h=1}^{H}\sum_{w=1}^{W}{\left|Y_{h,w}\right|}^{2}$.

The contrast metric reflects the clarity of high-frequency information in the image. The better focused the image, the greater the distinction between the target edges and the background, and the contrast value will also increase accordingly. The image contrast C in this paper is the ratio of the standard deviation of image intensity to the mean intensity.

(10) $$C=\frac{\sqrt{\left[{E\left({\left|Y\right|}^{2}-E\left({\left|Y\right|}^{2}\right)\right)}^{2}\right]}}{E\left({\left|Y\right|}^{2}\right)}$$

In the formula, $E\left[\cdot \right]$ represents the mathematical expectation (mean) operation over all pixels of the entire image. In the following experimental evaluation, we use these two indicators as benchmarks for measuring the ability of different algorithms to restore clear SAR images.

Peak Signal-to-Noise Ratio (PSNR) metric: It measures the reconstruction accuracy of an image by using the mean squared error between the focused image and a high-quality reference image. The higher the calculated PSNR, the smaller the difference for each pixel, meaning the image is better focused. Suppose the reference image has dimensions *H × W* with amplitude X, and the network outputs a focused image with amplitude |*Y*|, then the PSNR formula is as follows:

(11) $$\mathrm{PSNR}=10\;\log_{10}\left(\frac{{\mathrm{MAX}}_{X}^{2}}{\mathrm{MSE}}\right)$$

In the formula, ${\mathrm{MAX}}_{X}$ is the highest theoretical pixel value in the reference image; MSE is the mean squared error between the output image and the reference image, calculated using the following formula:

(12) $$\mathrm{MSE}=\frac{1}{H\times W}\sum_{h=1}^{H}\sum_{w=1}^{W}{\left(\left|Y_{h,w}\right|-X_{h,w}\right)}^{2}$$

In the formula, H and W represent the height and width of the image, respectively. $\left|Y_{h,w}\right|$ and $X_{h,w}$ correspond to the pixel values at the spatial coordinates (h,w) of the output focused image and the reference image.

The Structural Similarity Index (SSIM) evaluates the visual quality of a focused image by comparing the brightness, contrast, and structural information between the network output and the reference image. Unlike pixel-level error metrics, SSIM aligns more closely with human visual perception. The calculation formula is as follows:

(13) $$\mathrm{SSIM}=\frac{\left(2\mu_{\left|Y\right|}\mu_{X}+c_{1}\right)\left(2\sigma_{\left|Y\right|X}+c_{2}\right)}{\left(\mu_{\left|Y\right|}^{2}+\mu_{X}^{2}+c_{1}\right)\left(\sigma_{\left|Y\right|}^{2}+\sigma_{X}^{2}+c_{2}\right)}$$

In the formula, $\mu_{\left|Y\right|}$ and $\mu_{X}$ are the expected values (means) of the output image and the reference image, respectively; $\sigma_{\left|Y\right|}^{2}$ and $\sigma_{X}^{2}$ are their respective variances, and $\sigma_{\left|Y\right|X}$ is the covariance between them. $c_{1}$ and $c_{2}$ are tiny constants that help prevent numerical instability when the denominator gets close to zero.

### 3.4. Comparative Experiments

In this section, a performance comparison on the AFALOS test set is conducted. Specifically, the proposed algorithm is compared with other commonly used autofocusing methods. These include phase-gradient-based algorithms (PGA-ML, PGA-LUMV), minimum-entropy-criterion-based algorithms (NMEA), and deep learning-based networks (SAENet, AFnet, PAFnet). When reproducing SAENet, directly processing large-scale SAR images would greatly increase the number of parameters in the fully connected layers of the decoder, causing GPU memory bottlenecks; we therefore replaced them with 1 × 1 convolutions. This operation significantly reduces system memory consumption while also adhering to the physical principle that the original model’s decoder is a linear mapper. The experimental setup is as follows: 20,000, 8000, and 8000 images are taken from the AFALOS dataset to construct the training set, validation set, and independent test set, respectively. This part includes training the parameters that drive the network in this paper, determining the best weights, and then directly applying these weights in the testing phase.

To better quantitatively evaluate the focusing performance of different algorithms, Table 1 lists the average image entropy, contrast, PSNR, and SSIM of various autofocus algorithms on the AFALOS test set, where the metrics are recorded up to four decimal places to capture the subtle differences in fine-grained focusing performance. Generally, lower image entropy and higher contrast indicate that the defocused energy of the image has been more perfectly concentrated, and sidelobe interference is effectively suppressed. Meanwhile, higher PSNR and SSIM values directly reflect a greater degree of structural fidelity and detail restoration compared to the ideal focused reference. As can be seen from Table 1, compared with the uncorrected original images (Original), all autofocus algorithms can improve image quality to a certain extent.

Traditional PGA series algorithms (PGA-ML, PGA-LUMV) struggle to effectively extract strong scattering points in complex scenarios, resulting in minimal improvement in performance metrics. The iterative optimization-based NMEA algorithm provides clear advantages, but it is prone to getting stuck in local optima. Although SAENet incorporates a neural network architecture, it is essentially still a model-unfolding network based on self-supervised optimization from a single image. When facing nonlinear and spatial phase error problems, its feature perception capability is still inferior to deep feedforward neural networks trained on massive data (AFnet, PAFnet). The final quantitative comparison results show that the algorithm proposed in this paper achieves the best focusing performance among all methods. It not only achieved the lowest image entropy of 9.8567 and the highest contrast of 5.0091, but also a peak signal-to-noise ratio (PSNR) of 25.3870 dB and a structural similarity (SSIM) index of 0.9175, both being the best scores. The improvements in PSNR and SSIM indicate that the approach proposed in this paper is better at preserving scene structure and restoring object details.

This indicates that the range-aware attention mechanism designed in this paper can adaptively assign greater weight to range cells containing high-value strong scatterers, effectively suppressing the noise impact in homogeneous clutter areas and thereby providing purer features for the network to estimate azimuth phase errors. Besides, the simultaneous decrease in image entropy and increase in contrast is not accidental; it is an inevitable statistical result of the energy focusing in synthetic aperture radar. The network gathers scattered energy into sharp impulse responses, which inherently reduces intensity uncertainty (entropy decreases) while also increasing the grayscale difference between strong scatterers and the background clutter (contrast goes up).

**Figure 4:**
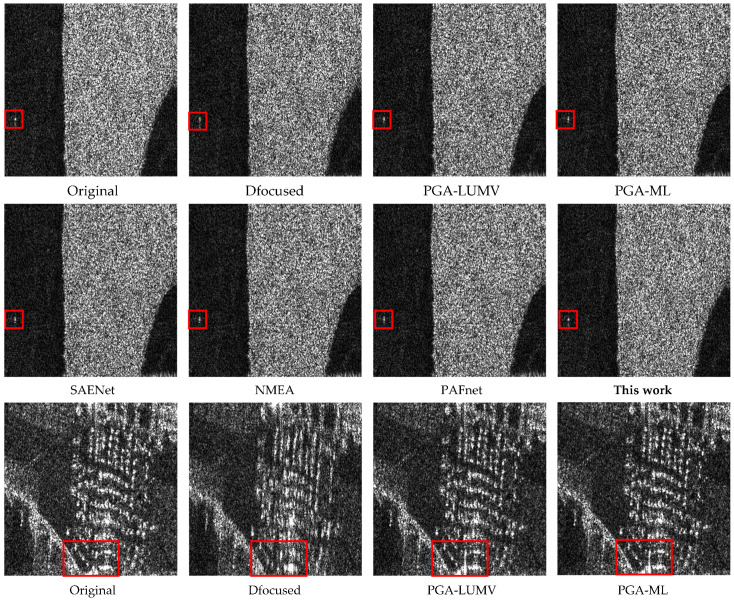

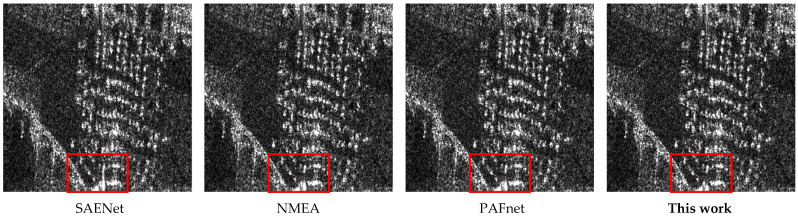
Focused SAR images of sparse scenes processed by different methods.

As shown in Table 2, deep neural networks (such as AFNET, PAFNET, SAENet, and our method) are all faster than traditional methods. On a GPU, our method can process 8000 images in just 13.86 s (roughly 1.73 milliseconds per image), which is nearly 4 times faster than NMEA and about 95 times faster than traditional PGA, completely breaking the computational bottleneck of classic autofocus algorithms and showing huge potential for real-time airborne or spaceborne deployment. To tackle the memory explosion caused by fully connected layers, we replaced SAENet’s fully connected layers with 1 × 1 convolutions, reducing the computational complexity from $O\left(N^{4}\right)$ to $O\left(N^{2}\right)$. The partial focused images obtained via diverse approaches are illustrated in Figure 4.

### 3.5. Ablation Study

To individualize and verify the quantitative impact of the deployed loss function and the range-aware attention module on the proposed network, a series of ablation studies were systematically conducted. The detailed experimental configurations and the corresponding numerical evaluation results are summarized and compiled in Table 3.

As compiled in Table 3, the baseline network yields an average image entropy of 9.8582 alongside a contrast of 4.9972. When purely configuring the multi-scale loss function, the image entropy decreases to 9.8573 while the contrast rises to 5.0031; similarly, when only embedding the range-aware attention module, the entropy drops to 9.8570 and the contrast escalates to 5.0063. These independent enhancements verify the individual efficacy of each respective modified component. Crucially, when concurrently fusing both modules, a prominent synergistic enhancement effect emerges: the high-weight sensitive features extracted by the attention mechanism achieve more sufficient parameter fitting under the precise constraints of the multi-scale loss function. This synergy drives the network’s focusing capability to its optimal state, securing the absolute minimum entropy of 9.8567 and the maximum contrast of 5.0091.

**Figure 5:**
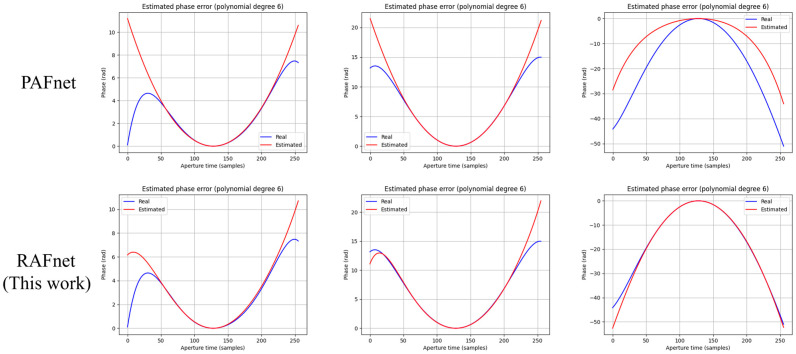
Phase error fitting curves.

To further explore the substantive impact of the aforementioned components from the dual perspectives of underlying physical mechanisms and network regression capability, Figure 5 intuitively compares the fitting profiles of the baseline model against the proposed methodology under complex high-order phase errors. In the graphical representation, the blue solid line traces the ground-truth phase error curve, whereas the red solid line maps the estimated curve generated by the network. The network does not directly output a high-dimensional phase sequence; instead, it reconstructs the estimated phase curve by regressing a set of polynomial coefficients. The mathematical model is as follows:

(14) $$\phi \left(t\right)=\sum_{q=2}^{Q}\alpha_{q}{\left(\frac{2t-N}{N}\right)}^{q}$$

where t is the aperture time sampling point, N is the total number of sampling points, Q is the highest order of the polynomial (set to 6 in this experiment), and $\alpha_{q}$ is the q-th order phase error coefficient of the network prediction. The validity of this polynomial approximation is constrained by the synthetic aperture duration, $t\in \left[0,N\right]$, within which the phase error remains within the model’s bandwidth limit.

It is clearly discernible that although the primitive baseline model manages to track the macroscopic trend of the phase error, a glaring residual deviation persists between the estimated and ground-truth curves when confronted with large-gradient variation regions (such as the aperture edges) or complex high-order polynomial perturbations. In sharp contrast, the proposed joint optimization framework exhibits an extraordinary capacity for phase tracking and profile fitting. Driven by the tight constraints of the multi-scale loss function and the energy concentration of sensitive features via the attention module, the red estimated curve nearly perfectly adheres to the blue ground-truth trajectory, maintaining exceptionally high fitting accuracy even at the extreme boundary points and sharp inflection locations on both ends of the curve.

Collectively, these findings substantiate the inherent soundness and efficacy of the proposed joint optimization strategy in enhancing high-precision SAR image autofocusing quality.

This article compares networks that embed the classic Squeeze-and-Excitation (SE) [43] channel attention mechanism, with quantitative results shown in Table 4. After adding the general SE channel attention, both the network’s peak signal-to-noise ratio (PSNR) and structural similarity (SSIM) show slight improvements, with PSNR increasing from 24.9399 dB to 25.0759 dB. SE slightly reduces image contrast, with the metric dropping from 4.9972 to 4.9936. The range-aware attention proposed in this paper achieves the best overall evaluation in terms of both image entropy and contrast, including the lowest image entropy (9.8570), the highest contrast (5.0063), the best PSNR (25.1797 dB), and the highest structural similarity (SSIM, 0.9142).

## 4. Discussion

As shown in the experiments, this method can offer significant benefits in performing operations with strong clutter. From a physical point of view, once the SAR data undergoes range cell migration correction (RCM correction), the overall compensation process is mathematically translated into 1-D azimuth autofocusing. However, if all range bins are treated equally while the feature aggregation is performed, then a massive number of pure clutter cells would inevitably be mixed into the phase estimator, which results in severe clutter coupling artefacts. The range-aware attention module acts as a data quality filter to suppress invalid clutter and amplify targets with a high signal-to-noise ratio (SNR) using range gating selection. The feature recalibration also suppresses interference terms. This can guarantee the reliability of phase estimation in difficult scenes. Although the proposed method proved capable of achieving high focusing performance in the current experiments, there are some obvious limitations to practical engineering applications. First, our model relies on the physical assumption of spatial phase error invariance in the local patches to perform its operation, thus lacking the decoupling capacity to efficiently resolve high-resolution and wide-swath-type phase anomalies. Second, while the range-aware attention module exploits feature discrimination and performs phase discrimination optimization, it also increases the time required for network forward inference.

Consequently, future research trajectories will be channeled into two primary thrusts: the first is to explore a space-time joint dual-stream attention mechanism in order to achieve high-precision 2-D autofocus; the second involves the incorporation of model lightweighting techniques, such as network pruning and knowledge distillation, specifically targeted at optimizing the attention module itself, thereby seeking an optimal trade-off between focusing accuracy and the real-time constraints of edge computing platforms.

## 5. Conclusions

Complex imaging scenes often introduce strong clutter as well as severe phase aberrations, degrading the performance of carrying out SAR autofocus. To address this, this paper introduces a range-aware attention mechanism to conduct feature enhancement. By relying on filtering out invalid clutter, the attention module successfully mitigates interference during the process of 1-D azimuth phase retrieval. Furthermore, a multi-scale autofocusing loss is implemented to prevent the optimization process from getting stuck in local minima, which is a common issue with traditional entropy metrics. Experiments utilizing real radar data confirm that this method yields better image entropy, contrast, and visual quality than conventional approaches. This study solves the 1-D autofocus problem for high-resolution SAR, providing a baseline for future extensions of the attention module to 2D autofocus. In addition, our future research will focus on building a multi-sensor complex SAR dataset to comprehensively evaluate and validate the cross-sensor generalization capability of the attention module proposed in this paper.

## Figures and Tables

**Table 1:** Quantitative comparison of different autofocusing methods.

Method	Entropy	Contrast	PSNR(dB)	SSIM
Original	10.0474	3.4077	13.5987	0.2732
PGA-ML	9.8913	4.7447	19.3876	0.7480
PGA-LUMV	9.8878	4.7726	19.9692	0.7611
SAENet	9.8821	4.7983	20.9549	0.7996
NMEA	9.8726	4.9604	21.6449	0.8396
AFnet	9.8616	4.9623	23.9827	0.8933
PAFnet	9.8582	4.9972	24.9399	0.9097
**This work**	**9.8567**	**5.0091**	**25.3870**	**0.9175**

**Table 2:** Running time comparison of different algorithms on 8000 test images.

Method	Total CPU Time (s)	Total GPU Time (s)	Avg. GPU Time per Image (ms)
PGA-ML	707.45	1309.64	163.71
PGA-LUMV	729.67	1312.77	164.10
SAENet	**410.61**	**3.17**	**0.40**
NMEA	967.11	52.67	6.58
AFnet	665.47	11.25	1.41
PAFnet	419.88	6.29	0.79
This work	495.77	13.86	1.73

**Table 3:** Ablation study on the proposed attention module and loss function.

Multi-Scale Loss	Attention	Entropy	Contrast
		9.8582	4.9972
✓		9.8573	5.0031
	✓	9.8570	5.0063
✓	✓	9.8567	5.0091

**Table 4:** Ablation study on different attention mechanisms.

Method	Entropy	Contrast	PSNR (dB)	SSIM
Baseline	9.8582	4.9972	24.9399	0.9097
Baseline + SE	9.8579	4.9936	25.0759	0.9115
Baseline + Ours	9.8570	5.0063	25.1797	0.9142

## Data Availability

ALOS SAR data were acquired from https://github.com/aisari/AutofocusSAR/tree/main/Dataset (accessed on 25 September 2025).

## Abbreviations

The following abbreviations are used in this manuscript:

SAR	Synthetic Aperture Radar
SNR	Signal-to-Noise Ratio
RCM	Range Cell Migration
RCMC	Range Cell Migration Correction
PGA	Phase Gradient Autofocus
MDA	Map Drift Autofocus
NMEA	Nonlinear Minimum Entropy Autofocus
LUMV	Linear Unbiased Minimum Variance
ML	Maximum Likelihood
